# Modeling and verifying clustering properties in a vehicular ad hoc network protocol with Event-B

**DOI:** 10.1038/s41598-021-97063-3

**Published:** 2021-09-02

**Authors:** Patrick Sondi, Imed Abbassi, Eric Ramat, Emna Chebbi, Mohamed Graiet

**Affiliations:** 1 Université Littoral Côte d’Opale, LISIC EA 4491, Calais, 62228 France; 2 Ecole Centrale de Lille, Cité Scientifique, BP 48, Villeneuve-d’Ascq, 59650 France; 3 University of Monastir, B.P 56, Avenue Taher Hadded, Monastir, 5000 Tunisia

**Keywords:** Engineering, Mathematics and computing

## Abstract

Vehicular ad hoc network (VANET) routing protocols resort to clustering in order to optimize broadcast traffic flooding. Clustering schemes usually rely on rules which apply to each vehicle in order to reach a targeted organization in a VANET. Most of the literature works which evaluate clustering for VANET focus on performance analysis. However, with autonomous vehicles coming to roadways, more rigorous relationships will be required between clustering rules and the resulting organization, so as to anticipate road safety in a better way. We propose a formal description of the properties which are expected in a VANET, while considering the rules of a given clustering scheme. Using Event-B, we first present a description of the VANET, the vehicles movement and the traffic generated by both routing and application messages. Then, based on an Event-B model of a basic routing protocol of the literature, we describe how the specific rules of a clustering scheme can be modeled along with the properties expected in the resulting organization. Finally, we propose a validation process of the model. This paper aims at showing how our proposals have been applied to the Chain-Branch-Leaf scheme, although they can be adapted to any rule-based clustering scheme for VANET.

## Introduction

### Context overview

Thanks to intelligent transport systems (ITS), new information and communication technology applications emerge in the transport sector and its logistics^1^. Vehicular Ad Hoc Network (VANET) extends ITS applications in a context where there is no available telecommunication infrastructure, in order to support road safety and cooperative applications through vehicle-to-vehicle communications^2^. One of the main issues VANET routing protocols must overcome is to achieve self-organization in the network, in a way which not only allows reducing the impact of flooding on the control traffic, but also favors network performance for the applications. For these reasons, clustering is one of the most important techniques used by routing protocols for vehicular ad hoc networks^3^. Particularly, the optimized link state routing (OLSR) protocol and its derivatives use multipoint relaying (MPR) as a clustering technique^4^. Each node selects a minimal subset of its one-hop neighbors which allows it to reach all the nodes located two-hop away. In this way, the multipoint relaying technique leads to a mesh between the vehicles covering the space in which they evolve. Several works show the very good results obtained thanks to MPR techniques for various ad hoc network based applications in open areas^5–7^. However, the MPR technique does not benefit from the particular configuration of road sections which are intrinsically spatially constrained. Chain-Branch-Leaf (CBL), a distributed clustering scheme exploiting this particularity, has been recently proposed^8^. It combines road configuration data, vehicle mobility and link quality indicators in order to build a structure similar to a vehicular network infrastructure, while relying only on vehicle-to-vehicle communications only. Thus, it organizes VANET nodes into a backbone of clusters (which is called “chain”), composed of branch nodes (cluster heads) to which leaf nodes (cluster members) attach themselves. Since 2018, up to thirty recent works referring to CBL have proposed similar mechanisms for various road traffic environments^9–12^. However, the evaluations presented in most of these works, which often rely on analytical or simulation models, focus on the performance of the clustering scheme in terms of ratio between the number of cluster head nodes and the total number of nodes, the number of backbones created in the network, and their impacts on routing overhead, packet delivery ratio, end-to-end delay... etc. Despite the accuracy of such evaluation regarding certain purposes, it is useful to evolve towards evaluation methods which allow providing more guarantees on the properties of a specific protocol or clustering scheme in a VANET environment. Formal methods (e.g., Petri nets^13^, finite state machines^14^, Event-B^15^, etc.) have proven their accuracy in the design of correct systems which properties can be proven using formal tools. They are based on mathematical foundations and first order logic to specify and perform reasoning about system properties. In this paper, we use the Event-B formal method to model and verify the properties of the CBL clustering scheme in order to show how such evaluation approach can be applied to VANET protocols.

### Related work

Several studies resort to formal modeling for the analysis and evaluation of ad hoc network routing protocols, especially regarding the functionalities which properties can be proven. Some works proposed a complete functional analysis of the protocols, notably DSR (dynamic source routing) protocol in Ref.^16^, AODV protocol (Ad hoc On-demand Distance Vector) in Ref.^17^ and OLSR (Optimized Link State Routing) protocol in Ref.^18^. These analysis particularly focus on network link detection, route discovery, and relay selection (only for OLSR). However, the spatial relations between the network nodes, and their movement, which are particularly important in VANET, were not considered in these works. Other proposals focus on specific properties of the mechanisms proposed by routing protocols for ad hoc networks. The work in Ref.^19^ introduced a series of proposals for the modeling and evaluation of quality of service in communication protocols, using formal methods and languages. Similar proposals were presented for the verification of security properties in ad hoc routing protocols^20,21^. A more recent work^22^ also proposes a complete analysis of the Zone Routing Protocol (ZRP), including a detailed description of its formal model and its verification and validation process using Event-B. However, in ad hoc networks the routing protocol is also at the heart of the self-organization of the network. Particularly, in vehicular ad hoc networks, the spatial relations and the movement of the vehicules directly impact that organization. These aspects were not considered in the formal modeling proposed in the previous works. Recently, we presented a preliminary Event-B model for the Chain-Branch-Leaf (CBL) clustering scheme^23^ which we proposed for achieving self-organizing in a model of OLSR protocol inspired from^18^. The present work is in keeping with it, but instead of simply extending the previous model^23^, it proposes an original model of CBL independently from its implementation in OLSR, and analyses its correctness regarding the specifications of CBL rules. Moreover, it describes how we modeled the spatial relations between the vehicles, their movement, and also network topology discovery and routing functions that are common to most routing protocols for VANET, which could be reused in order to evaluate any other clustering scheme.

The rest of this paper is organized as follows: “Resuts” section describes CBL functioning and a correct-by-construction model for CBL (*CCM*4*CBL*); the method used during the consistency verification process of the proposed model is described in “Discussion and methods” and “Conclusion and prospective work” section presents our conclusion and prospective for future work.

## Results

This section presents the main result of this work, which is a formal model of the Chain-Branch-Leaf clustering scheme allowing verifying its properties in a VANET. As for every clustering schemes, several rules are taken into account by the vehicles involved in the construction of both the clusters and the overall backbone. For example, the uniqueness of the branch node choice for each leaf node is a fundamental property of the Chain-Branch-Leaf organization^24^. However, without a formal description of the CBL scheme, such a requirement can only be envisioned, but not formally expressed nor verified. The assessment of the CBL clustering scheme implies two steps, which are requirement validation for verifying that the protocol specification fulfills the functional needs envisaged by the designer^25^, and consistency checking for ensuring that the clustering scheme does not introduce contradictions in the relations linking the nodes. Event-based modeling is particularly suitable for protocol engineering. Event-B is precisely an event-based formal method which has shown its capacity to master the system design complexity through successive refinements^15,26^. The stepwise refinement produces a correct-by-construction model by formally proving the different properties introduced up to each step^26^. Regarding CBL, Event-B provides the tools necessary to perform an incremental verification by checking the properties and constraints defined at each execution step. The different execution steps are characterized by the introduced events. In order to guarantee the invariants preservation by these events, Event-B defines the concept of proof obligation. Therefore, the approach proposed in this paper should allow checking and proving the correctness of the CBL protocol as well as its requirements and properties. Then, once the CBL protocol has been verified, the proposed model guarantees that its execution does not face failure or inconsistency.

### Formal description of CBL organization in a VANET

**Figure 1:**
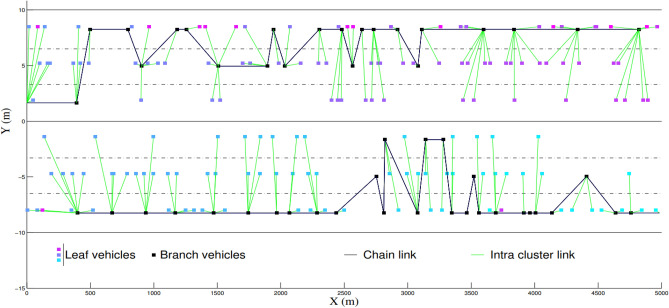
Example of two CBL chains on a 2-way highway.

In this section, we first describe CBL functioning along with the related formal definitions. CBL is a distributed algorithm performed by each VANET node^27^, considering that the latter may not have a global knowledge of the ad hoc network. Thus, a node can only communicate directly only with its one-hop neighbors which are used as routers in order to reach the rest of the network. A complete description of CBL functioning is presented in Ref.^24^. Figure 1 shows the different elements of the CBL structure in a VANET, which can be summarized as follows:

- *Road configuration* in any road configuration, CBL builds one backbone in each traffic direction. In our example, a 3-lane 2-way highway, CBL builds two separate backbones (Fig. 1).
- *A branch node* is a cluster-head node elected by other nodes (branch or leaf). It is the only one allowed to retransmit the broadcast traffic to the entire network, through its downstream branch, its upstream branch or both.
- *A leaf node* is an ordinary node which attaches itself to the closest branch node. If no branch node is detected, the leaf nodes perform a branch choice process in order to elect one of them.
- *A chain* is a virtual backbone made up of a sequence of branch nodes. Ideally, one chain per traffic direction should be created. On a longitudinal road context such as highways, a chain behaves as a virtual backbone similar to that which should be obtained with a fixed infrastructure. It provides branch nodes with a path for more than one-hop communications.

In this work, the term *Nodes* refers to the set of all the nodes in the network. Without loss of generality, we will focus in this paper on two types of packets which are sufficient to describe CBL functioning. The hello packets are used by several VANET protocols for neighborhood discovery. The other type of packet refers to the applications traffic. The term *Hello* refers to the set of all the hello packets sent during the VANET scenario. We formally define VANET links using the following functions:

1 $$\begin{aligned}&links\in {}\mathbb {P}(Nodes\mathbin \times {}Nodes), \end{aligned}$$

2 $$\begin{aligned}&links\cap {}(Nodes\mathbin \lhd {}id) = \mathord \varnothing {}. \end{aligned}$$

An element $n1\mapsto {}n2$ of the set *links* ($n1\mapsto {}n2 \in {} links$) expresses that the node *n*1 has received a hello packet sent by the node *n*2. We formally define the local neighbors of nodes using the following function:

3 $$\begin{aligned} neighbors\in {} Nodes\mathbin \rightarrow {}\mathbb {P}{}(Nodes). \end{aligned}$$

For a given node *n*, the following property must be fulfilled:

4 $$\begin{aligned} \{n\}\mathbin \times neighbors(n)\subseteq \{n\}\mathbin \lhd {}links. \end{aligned}$$

One of the main data required by CBL is node position. We assume that each node is aware of its position through a global positioning system such as GPS or Galileo. However, we avoid any terrestrial localization infrastructure since ad hoc networks should not rely on any infrastructure. The node’s position is a two-dimensional vector, that the node transmits to its neighbors through hello packets. We formally model the nodes positions data using the following function:5

Let us consider a node *n*, the set *positionTable*(*n*) includes the position of *n* and those of its local neighbors. A node can be positioned in the downstream or the upstream side of any of its neighbors. We formally define the downstream/upstream neighbors of nodes using the following functions:

6 $$\begin{aligned}&down \in {} Nodes\mathbin \rightarrow \mathbb {P}(Nodes), \end{aligned}$$

7 $$\begin{aligned}&up \in {} Nodes\mathbin \rightarrow \mathbb {P}(Nodes). \end{aligned}$$

To determine whether a node is upstream or downstream from another, we use the following operator:

$$\begin{aligned} DIFF=(\lambda {}(a\mapsto {}b)\mapsto {}(c\mapsto {}d)\cdot {}\{a,b,c,d\}\subseteq \mathbb N|(a--c)). \end{aligned}$$

Let *n*1 and *n*2 be two neighboring nodes, and let p1 and p2 be their respective positions. We state the following rules

- *n*2 is an upstream node of *n*1 if $DIFF(p1 \mapsto {} p2)$ is positive.
- *n*2 is a downstream node of *n*1 if $DIFF(p1 \mapsto {} p2)$ is negative.

When a node does not receive any hello message from a neighbor within a specific period of time, this neighbor is considered to be unavailable and it will be deleted from the neighbors table. This time interval called neighbor expiry time is defined as:

8 $$\begin{aligned} Neighbor\_expiry\_time\in {}\mathbb N{}. \end{aligned}$$

In the CBL clustering scheme, a node can be either a leaf or a branch node. We formally define the node types as follows:9

A branch node *n* ($hasType(n)(n)=1$) is a cluster-head node elected by the other nodes in its one-hop neighborhood. It emits hello messages like every node. A leaf node *n* ($hasType(n)(n)=0$) is an ordinary node which has to connect to the closest branch node. A CBL chain is a sequence of branch nodes. We formally define this chain of branch nodes as follows:

10 $$\begin{aligned}&chainUP \in {} Nodes\mathbin \rightarrow {}\mathbb {P}(Nodes \mathbin \times {} Nodes), \end{aligned}$$

11 $$\begin{aligned}&chainDO \in {} Nodes\mathbin \rightarrow {}\mathbb {P}(Nodes \mathbin \times {} Nodes). \end{aligned}$$

These two functions are semantically opposed. Hence, *chainUP* (resp. *chainDO*) function defines a local upstream (resp. downstream) chain of branch nodes. If a branch b2 is an upstream node of another branch b1, then b1 is a downstream node of b2. In a CBL organization, each leaf node shall elect its associated branch. We formally define this election as follows:

12 $$\begin{aligned} branchChoice \in {} Nodes\mathbin \rightarrow {}\mathbb {P}(Nodes \mathbin \times {} Nodes). \end{aligned}$$

For a given branch node *n*, branchChoice(n), chainUP(n) (resp. chainDO) refer to the branch choice (if the electing node is a leaf node) or the upstream (resp. downstream) branch nodes (if the electing node is a branch node) included in the neighbors table of *n*. A hello packet can contain different data about its sender, namely the node’s position, the node’s type, and according to the latter, the elected branch up/down, or the branch choice. To formally define all these data, we use the following functions:1314151617

For a given hello packet *h* sent by a node *n*, *helloPosition*(*h*), *helloBrChoice*(*h*), *helloChainUP*(*h*), *helloChainDO*(*h*) and *helloSrType*(*h*) represent respectively the sets of the position, branch choice, elected branch up, elected branch down and the type of *n* and its neighbors. All these data are very useful in the construction of the CBL scheme. The connection time is the expected communication duration of two nodes according to their movement. More formally, we define the following function:18

### CBL properties and rules

Now that CBL organization has been clarified, it is possible to express the expected properties, and the rules established to that end. The main requirements and resulting properties expected from CBL organization in a VANET are:

- *REQ 1* If a node does not have any local neighbor, it must be a leaf.
- *REQ 2* The branch node choice is made according to leaf nodes only.
- *REQ 3* The self-branch election is not possible.
- *REQ 4* Each branch shall have at most one downstream branch neighbor.
- *REQ 5* Each branch shall have at most one upstream branch neighbor.
- *REQ 6* Each branch shall be elected by at least another node (branch or leaf).
- *REQ 7* A node having a downstream branch shall be of type branch.
- *REQ 8* The electing node of an upstream branch shall be a branch-type one.
- *REQ 9* If a node *n*1 is a downstream branch of a node *n*2, then *n*2 is an upstream branch of *n*1.
- *REQ 10* An upstream branch of a node shall be one of its upstream neighbors.
- *REQ 11* A downstream branch of a node shall be one of its downstream neighbors.
- *REQ 12* One of the neighbors of each leaf node shall become a branch.

Different constraints shall be satisfied while electing branch nodes, such as the following node election rules (NER):

- *NER1* The self-node election is not possible (i.e., the elected node and the electing one must be different).
- *NER2* The elected node must be a neighbor of its electing node.
- *NER3* The elected node must be an upstream neighbor of its electing branch node.
- *NER4* A branch node must have at most one upstream neighbor branch.
- *NER5* When the electing node is a leaf node which does not have any branch node neighbor, it must elect the leaf node neighbor having the maximum connection time as its branch choice.
- *NER6* When the electing node is a branch node which does not have any upstream branch node neighbor, it must elect the upstream neighbor node having the maximum connection time as its upstream branch node.

All nodes are initially leaf nodes. Some of the nodes can be turned into branch nodes, while others must be kept as leaf nodes. In addition, a branch can be turned into a leaf. The type changing rules (TCR) are the following:

- *TCR1* If a leaf node is elected by another node, it must be turned into a branch.
- *TCR2* If the electing node is a branch, then it shall be added to the chain as a downstream branch of the elected one.
- *TCR3* The branch node which overtakes its downstream electing branch shall be turned into a leaf.

### A correct-by-construction model of CBL (CCM4CBL)

**Figure 2:**
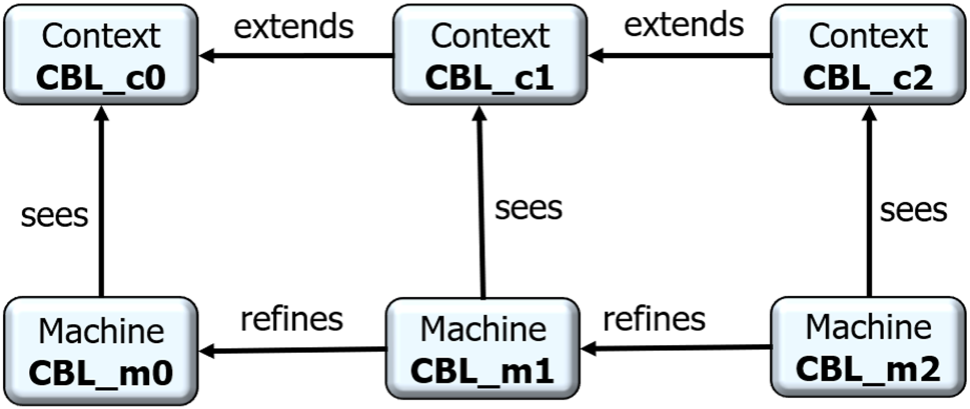
Architecture of the Event-B model for CCM4CBL.

In this section, is presented the proposed Event-B model of the CBL clustering scheme, which implements the aforementioned properties and rules (see “CBL properties and rules” section). Figure 2 illustrates the architecture of the resulting model. It shows three abstraction levels which will be detailed in the following subsections. The two first levels can be used for modeling any other VANET routing protocol.

#### Level 1: a basic routing protocol model (CBL_c0, CBL_m0)

**Figure 3:**
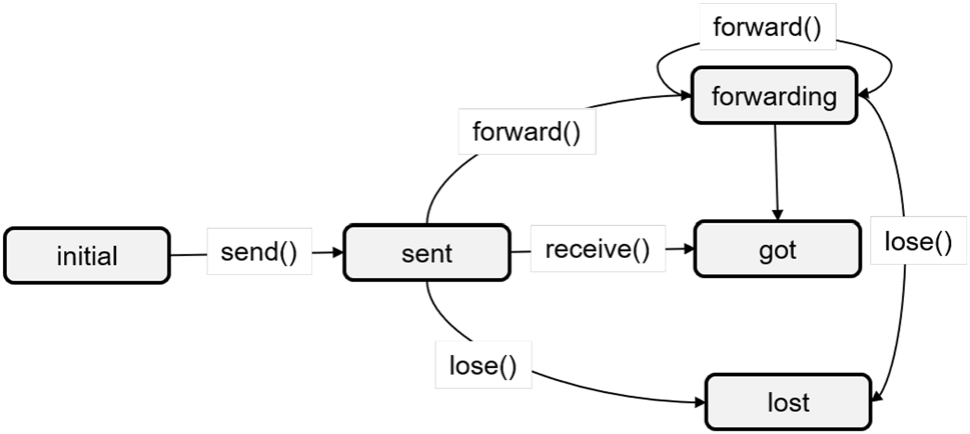
Statechart of packet transfer process.

**Figure 4:**
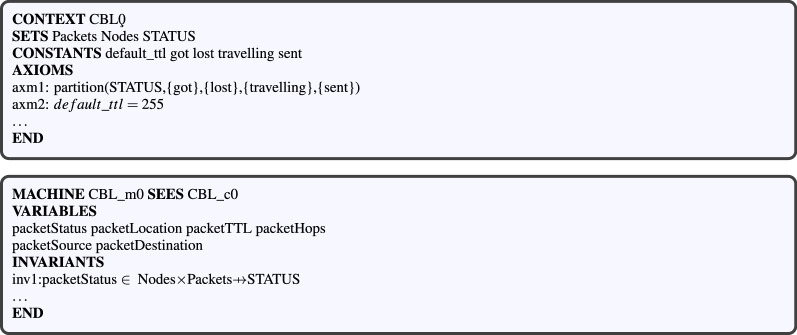
Level 1: nasic protocol model including a machine and a context.

This model inspired from Ref.^18^ is an Event-B formal model of a basic routing protocol. It includes the definitions of the set of nodes, that of the links, and also the events related to packet status and operations (Fig. 3). It can be extended and refined in order to model any other routing protocol. Although we rewrote the model (Fig. 4), a similar one is available in the literature^23^. This model has been implemented through the contex *CBL_c0* and the machine *CBL_m0* illustrated in Fig. 2.

#### Level 2: modeling VANET node dynamics and communications (CBL_c1, CBL_m1)

**Figure 5:**
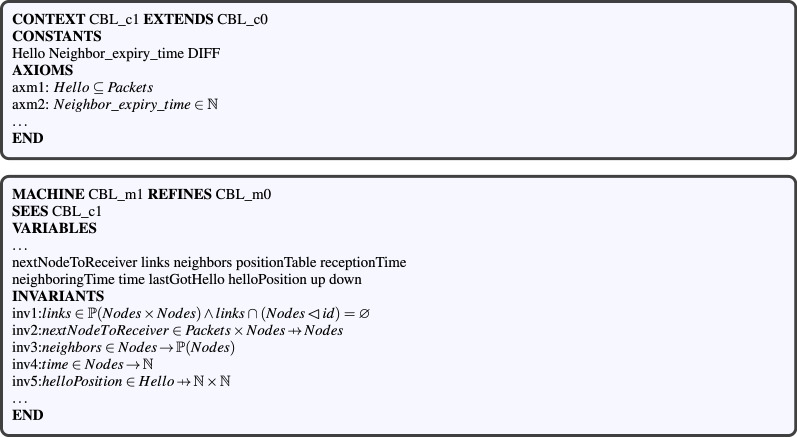
Level 2: modeling VANET nodes dynamics and communications.

This second abstraction level of the *CCM*4*CBL* model formally defines the concepts related to VANET node dynamics and communications, notably neighborhood management, nodes positioning in the road traffic, and packet broadcasting. It consists in the $CBL\_m1$ machine and the $CBL\_c1$ context (see Fig. 5). In addition to the definitions in “Formal description of CBL organization in a VANET” section, the $CBL\_c1$ context extends the initial $CBL\_c0$ context by introducing the finite set of all the hello packets ($Hello \subset Packets$). Given the $CBL\_c1$ context, the $CBL\_m1$ machine refines $CBL\_m0$ by introducing the variables, invariants and events modeling VANET node communications, vehicles movement in the road traffic, and neighborhood discovery and management.

#### Modeling VANET node communications

**Figure 6:**
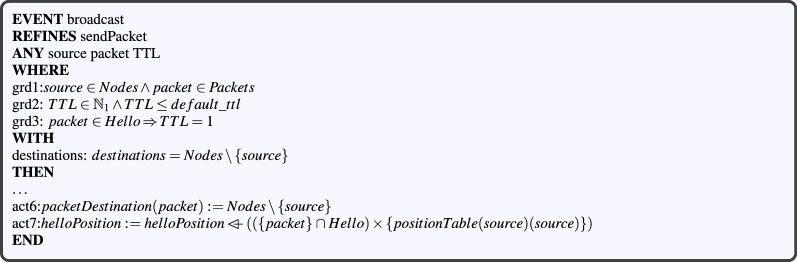
The broadcasting packet event.

Communications in a VANET not only include the forwarding of application packets, but also the broadcasting of hello packets for neighborhood discovery and link detection. To formally model ad hoc communications in a VANET, in addition to the definitions proposed in “Formal description of CBL organization in a VANET” section, we introduce the following variables :

- *nextNodeToReceiver* a variable determining the next receiver node of each sent packet (see *inv*2, Fig. 5). This variable is used for forwarding packets from its source to its destination.
- *lastGotHello*: a variable determining the last hello a node received from another. We define this variable as follows:
- *time* a variable determining the local time of each node (see inv4, Fig. 5)^24^.
- *receptionTime* A variable determining the reception time of a packet according to its destination node. We formally define this variable using an invariant as the following function:
- *neighboringTime* a variable determining the time when a node becomes another’s neighbor. We formally define this variable as the following function:

These variables *time*, *receptionTime* and *neighboringTime* are mainly used in order to control the availability of a node’s neighbors, as it will be detailed in the neighborhood management phase. The *sendPacket*, *forwardPacket* and *receivePacket* abstract events are also refined. Two refined versions are proposed for the packet sending event, namely sendPacket and broadcast. The first one is used for sending application packets, while the second allows broadcasting of some specific packets such as hello. As Fig. 6 shows, the abstract parameter *destinations* has disappeared from the packet broadcasting event, while new guards and actions have been added. In addition, a witness clause (*with*) is included in order to define the link between the abstract event and the refined one. This witness states that broadcast packets are able to be received by any node in the ad hoc network, with the exception of the sender. This requires a replacement of every occurrences of *destinations* in the action clause with $Nodes\setminus {}\{source\}.$ Action *act*6 illustrates an example of such replacement. Guard *grd*3 ensures that the TTL of a Hello-type packet is equal to 1 during the broadcast process, in order to avoid its forwarding. Action *act*7 expresses that the hello packet sender shall broadcast its current position to all its neighbors. A new parameter *T* representing the local time at the destination node, and new guards are added in the refined version of the packet receiving event. The guard clause is extended with the following three predicates:

- $$(packet\notin {}Hello \mathbin \Rightarrow {} packetLocation(destination \mapsto {} packet)\mapsto {}destination \in {}cls(links)), $$
- $$T\in {}\mathbb N{}\wedge {}time(destination)\le {}T,$$
- $$(packet\in {}Hello\mathbin \Rightarrow {}destination\mapsto {}packet\mapsto {}travelling\notin {}$$*packetStatus*),

where *cls* refers to the mathematical closure operator. For a relation $R\in A\mathbin \leftrightarrow A$, *cls*(*R*) is the closure of *R*, which we define as follows: $cls(R)=\bigcup _{i=0}^{\infty } R^i$, where

$$\begin{aligned} R^i={\left\{ \begin{array}{ll} A \triangleleft id, &{} \text {if }i=0\\ (R \mathbin ;R^{i-1}), &{} \text {if } i\ge 1. \end{array}\right. } \end{aligned}$$

The actions added in the refinement of the reception event, update *time*, *receptionTime* and *lastGotHello* variables. In the refined packet forwarding event (*forwardPacket*), we introduce a new parameter *nextNode* (nextNode $\in {}$ Nodes), new guards and a new action. The added guards are used to define the connection links between a packet’s source/destination and the forwarding node, while the action updates the nextNodeToReceiver variable as follows:

#### Modeling the vehicle movement

**Figure 7:**
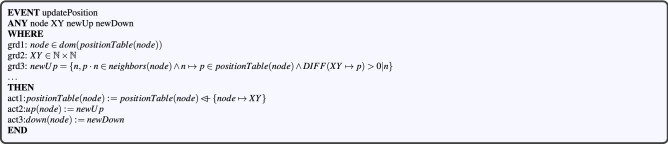
Updating vehicles position event.

An *updatePosition* event is defined in the $CBL\_m1$ machine in order to model the movement of vehicles (described as members of the set Nodes in our model) on the road. As Fig. 7 shows, this event takes the following parameters as input:

- *node* represents a vehicle whose position shall be updated.
- $XY\in {}\mathbb N{}\mathbin \times {}\mathbb N{}$ refers to the new position of the node.
- *newUp* and *newDown* denote the updated upstream and downstream neighbors of the node.

The last two parameters are checked automatically based on the new node’s position using *grd*3 and *grd*4 guards. The event updates the *positionTable* variable by overriding the *positionTable*(*node*) with a set composed of the element $\{node\mapsto {}XY\}$. The *act*2 and *act*3 actions respectively update the upstream (*newUp*) and downstream (*newDown*) neighbors of the node.

#### Modeling local neighborhood management

**Figure 8:**
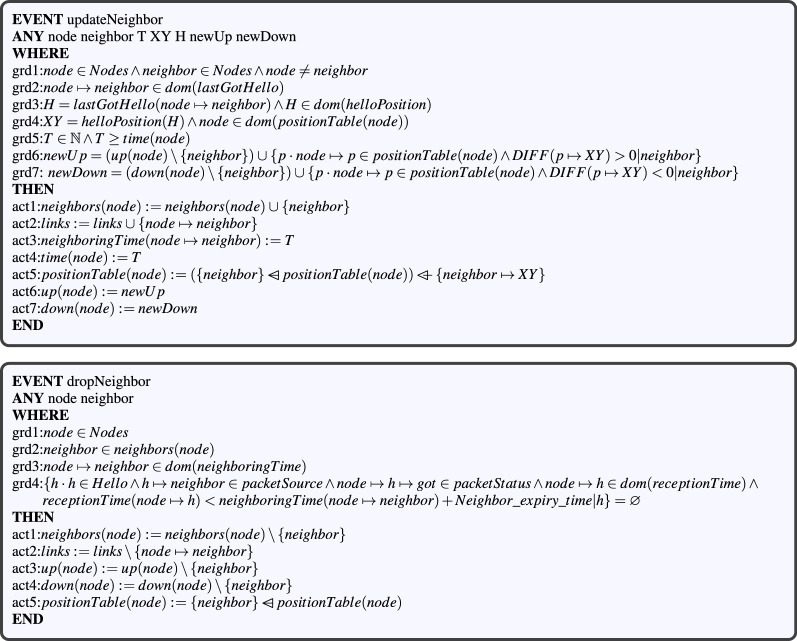
The neighbor updating/removing events.

When a node receives a hello packet, it must update its neighbors table according to its content. To model this formally, we add an *updateNeighbor* event in the $CBL\_m1$ machine (see Fig. 8). This event has six parameters, three of which (*newUp*, *newDown* and *H*) being automatically computed through the guards, based on the first four parameters. The first two parameters are the source (*neighbor*) and its position *XY* contained in the last hello *H* received from it. Parameters *H* (the last received hello), *newUp* (the updated upstream nodes) and *newDown* (updated downstream nodes) are merely used for simplification. The other two parameters are a destination *node* and its current time *T*. The above parameters are typed through the guards. As stated in Ref.^24^, each node periodically broadcasts hello packets to declare its availability to all its neighbors. Thus, when a node does not receive a hello packet from a neighbor within a period of time which equals to the $neighbor\_expiry\_time$, this neighbor is considered to be unavailable and is deleted from the node’s neighbors table. More formally, we create a *dropNeighbor* event in the $CBL\_m1$ machine (see Fig. 8). As input parameters, the *dropNeighbor* event has a node and its unavailable neighbor. These parameters are well-defined by *grd*1, *grd*2 and *grd*3 guards. The *grd*3 guard checks the unavailability precondition of the neighboring node before applying the necessary actions.

#### Level 3: modeling CBL properties and rules (CBL_c2, CBL_m2)

**Figure 9:**
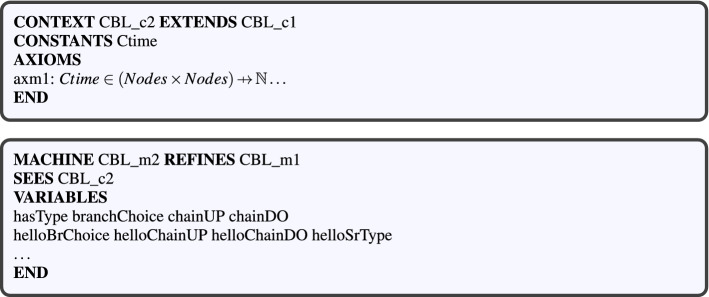
Level 3: modeling CBL properties and rules.

The last abstraction level of our CCM4CBL model introduces the specific properties and rules of the CBL clustering scheme in a VANET. As Fig. 9 shows, this implementation level consists in a $CBL\_m2$ machine which sees a $CBL\_c2$ context.

**Figure 10:**
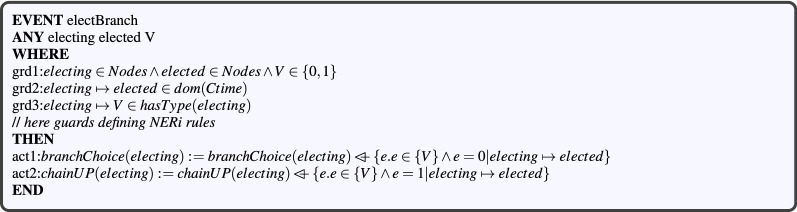
Branch election event.

The $CBL\_c2$ context extends the context of the second level ($CBL\_c1$) by introducing the concept of node connection time (*Ctime*). The latter is axiomatically defined according to the definition proposed in “Formal description of CBL organization in a VANET” section. Figure 9 also depicts a machine which models CBL properties and rules. This machine sees the $CBL\_c2$ context, and refines $CBL\_m1$ machine. The presentation of the refinements is organized in four steps, which are: (1) modeling CBL properties, (2) modeling branch nodes election, (3) refining ad hoc communications in a VANET, and (4) modeling CBL chain update.

**Table 1:** Formal modeling of CBL properties with Event-B as invariants.

Property	Corresponding Event-B invariant
REQ 1	$$\forall {}n\cdot {}n\in {}Nodes\wedge {}neighbors(n)=\mathord \varnothing {} $$$$\mathbin \Rightarrow {} n\mapsto {}0\in {}hasType(n)$$
REQ 2	$$\forall {}n,a\cdot {}n\in {}Nodes\wedge {}a\in {}dom(branchChoice(n)) $$$$\mathbin \Rightarrow {} a\mapsto {}0\in {}hasType(n)$$
REQ 3	$$\forall {}n\cdot {}n\in {}Nodes $$$$\mathbin \Rightarrow {} (chainUP(n)\mathbin {\cup }{}branchChoice(n))\mathbin {\cap }{}(Nodes\mathbin \lhd {}id)=\mathord \varnothing {}$$
REQ 4	
REQ 5	
REQ 6	$\forall {}n\cdot {}n\in {}Nodes\wedge {}n\mapsto {}1\in {}hasType(n) \mathbin \Rightarrow {}(chainUP(n)\mathbin {\cup }{}branchChoice(n))\mathbin \rhd {}\{n\}\ne {}\mathord \varnothing {}$
REQ 7	$$\forall {}a,n\cdot {}a\in {}Nodes\wedge {}a\mapsto {}n\in {}chainDO(n)$$$$\mathbin \Rightarrow {}a\mapsto {}1\in {}hasType(n) $$
REQ 8	$$ \forall {}a,n\cdot {}a\in {}Nodes\wedge {}a\mapsto {}n\in {}chainUP(n)$$$$\mathbin \Rightarrow {}a\mapsto {}1\in {}hasType(n) $$
REQ 9	$$ \forall {}a,n\cdot {}a\in {}Nodes\wedge {}a\mapsto {}n\in {}chainUP(n)$$$$\mathbin \Rightarrow {}a\in {}down(n) $$
REQ 10	$$ \forall {}a,n\cdot {}a\in {}Nodes\wedge {}a\mapsto {}n\in {}chainDO(n)$$$$\wedge {}n\mapsto {}a\in {}chainUP(n) $$$$ \mathbin \Rightarrow {}a\in {}up(n) $$
REQ 11	$$ \forall {}a,n\cdot {}a\in {}Nodes\wedge {}a\mapsto {}n\in {}chainDO(n)\wedge {}a\in {}up(n)$$$$\mathbin \Rightarrow {}n\mapsto {}a\in {}chainUP(n) $$
REQ 12	$$ \forall {}n,b\cdot {}n\in {}Nodes\wedge {}n\mapsto {}b\in {}branchChoice(n)$$$$ \mathbin \Rightarrow {} b\in {}neighbors(n) $$

#### Modeling CBL properties

New variables are introduced in the $CBL\_m2$ machine in order to formally model the specific properties and rules of CBL:

- *hasType* a variable determining the type of the node (Branch or Leaf).
- *branchChoice* a variable determining the branch choice of the node.
- *chainUP* a variable determining the upstream branch node.
- *chainDO* a variable determining the downstream branch node.

The type of each variable is defined using a typing invariant according to the definitions introduced in “Formal description of CBL organization in a VANET” section. Given the variables, constants and sets introduced, CBL properties (*req*1, *req*2, $\ldots$, *req*12) can be formally expressed as invariants in Event-B. These invariants are illustrated in Table 1.

#### Modeling branch nodes election

This election is a key step in the construction of the CBL structure. As stated in Ref.^24^, two election types are possible: branch choice by leaf nodes, and upstream branch election by branch nodes. To model these two operations formally, we created an *electBranch* event, taking three parameters as input (see Fig. 10). The first two parameters, *electing* and *elected*, refer to both the electing and elected nodes. The other parameter, *V*, is a binary number ($V\in {}\{0,1\}$) used to simplify the verification of the *NERi* rules while electing branch nodes. To achieve this verification, each *NERi* rule is defined as a guard in the *electBranch* event. Each one of these guards is detailed below. The first rule, *NER*1, states that a node (leaf or branch) cannot elect itself as a branch, while the second one (*NER*2) stipulates that the elected node must be a neighbor of its electing one. These two rules are defined as follows :

$$\begin{aligned} elected\ne {}electing\wedge {}elected\in {}neighbors(electing)\wedge {}~electing\mapsto {}0\in {} hasType(electing). \end{aligned}$$

Rule *NER*3 states that the elected node must be an upstream neighbor of its electing node, which shall be a branch. This rule is expressed through the following predicate:

$$\begin{aligned} electing\mapsto {}1\in {}hasType(electing)~ \mathbin \Rightarrow {} elected\in {}up(electing). \end{aligned}$$

Rule *NER*4 is used to ensure the uniqueness of the CBL chain, in order to avoid parallel chains in the same area. Hence, it expresses that a branch node must have at most one upstream neighbor branch. We define this rule as follows:

$$\begin{aligned} V=1 \mathbin \Rightarrow {} chainUP(electing)\mathbin \rhd {}up(electing)=\mathord \varnothing {}. \end{aligned}$$

Rule *NER*5 states that, when there is no neighboring branch node, the electing leaf must choose the neighbor having the maximum connection time to itself. We formally define this constraint through the following predicate:

$$\begin{aligned}&elected\mapsto {}0\in {}hasType(electing)\wedge {}V=0\\&\quad \mathbin \Rightarrow {} \lnot {}(\exists {}n\cdot {}n\in {}neighbors(electing) \wedge {}electing\mapsto {}n\in {}dom(Ctime)\wedge {} Ctime(electing \mapsto {}elected)<Ctime(electing\mapsto {}n)). \end{aligned}$$

Rule *NER*6 concerns the connection time of the elected upstream branch. This rule is applied when the electing branch node does not have any upstream branch-type neighbor. It is formally defined similarly to rule *NER*5 as follows:

$$\begin{aligned}&\{elected\mapsto {}0\}\subseteq {}hasType(electing)\wedge {}V=1\\&\quad \mathbin \Rightarrow {} \lnot {}(\exists {}n\cdot {}n\in {}up(electing)\wedge {} electing\mapsto {}n\in {}dom(Ctime)\wedge {} Ctime(electing \mapsto {}elected)<Ctime(electing\mapsto {}n)). \end{aligned}$$

#### Refining VANET node communications

As stated previously, CBL is a distributed algorithm which builds a backbone of branch nodes, and clusters of leaf nodes around the latter. In order to achieve this task, CBL relies only on the information exchanged through hello packets. A hello packet contains information about its sender, such as its position, its type (branch or leaf), its branch choice when it is a leaf node, its upstream and downstream branch nodes when it is a branch node, and its direct neighbors. The following variables are added:

- *helloBrChoice* a variable determining the branch choice of the hello sender.
- *helloChainUP* a variable determining the upstream branch of the hello sender.
- *helloChainDO* a variable determining the downstream branch of the hello sender.
- *helloSrType* a variable determining the type of the hello sender (branch or leaf).

These variables are defined as partial functions using invariants in the $CBL\_m2$ machine according to their definition (see “Formal description of CBL organization in a VANET” section). The packet broadcasting event is refined in order to integrate the information contained in the hello packet. A guard related to CBL property *REQ*12 is added in order to enforce the leaf nodes which have neighbors, so that they can choose their branch nodes before broadcasting their hello packet:

$$\begin{aligned} neighbors(source)\ne {}\mathord \varnothing {} \wedge {}source\mapsto {}0\in {}hasType (source) \mathbin \Rightarrow {}~1\in {}hasType(source)[neighbors(source)]. \end{aligned}$$

New substitution actions are also introduced in the refined version of the *broadcast* event, in order to update the status of the *helloBrChoice*, *helloChainUP*, *helloChainDO* and *helloSrType* variables as follows:

#### Modeling CBL chain update

Several events trigger the update of the CBL chain locally at the VANET node level, such as the unavailability of some neighbors, or the overtaking of a branch node by its upstream branch node. In this work, we consider three events, namely: a neighbor which is no longer available, changes in the nodes’ positions, and a received hello packet indicating some changes in the CBL chain (election of new branch up/down, branch choice, etc.). In order to model these events formally, we refine *updatePosition*, *dropNeighbor* and *updateNeighbor* abstract events, and introduce two new events, *turnIntoBranch* and *turnIntoLeaf*, which are used to change the node type according to TCR rules (“CBL properties and rules” section). The *updateNeighbor* variable is refined as follows:

$$\begin{aligned} hasType(node)&:={}newTypes \\ chainDO(node)&:={}newChainDO \\ chainUP(node)&:={}newChainUP \\ branchChoice(node)&:={}newBrChoice \end{aligned},$$

where *newTypes*, *newChainDO*, *newChainUP* and *newBrChoice* refer to the parameters introduced in the event’s refinement, and are the new values of functions *hasType*(*node*), *chainDO*(*node*), *chainUP*(*node*) and *branchChoice*(*node*) after updating or adding the information (type, branch choice, and so on) about the neighbor. For example, the values of *newChainUP* and *newChainDO* parameters are computed as follows:

The other parameters are computed similarly. The *dropNeighbor* refined event allows the local update of the CBL chain in case of unavailability of a neighbor. Compared to its abstract version, it introduces the following actions:

Updating the position of a node *Ni* requires checking whether *Ni* has overtaken the node *Nj* that elected it as its upstream branch node. In this case, the chain link between *Ni* and *Nj* must be deleted by adding an action in the *updatePosition* event:

$$\begin{aligned} chainUP(node):={} newChainUP \end{aligned}$$

where *newChainUP* is a new parameter referring to the updated upstream branch node, automatically computed as follows:19

**Figure 11:**
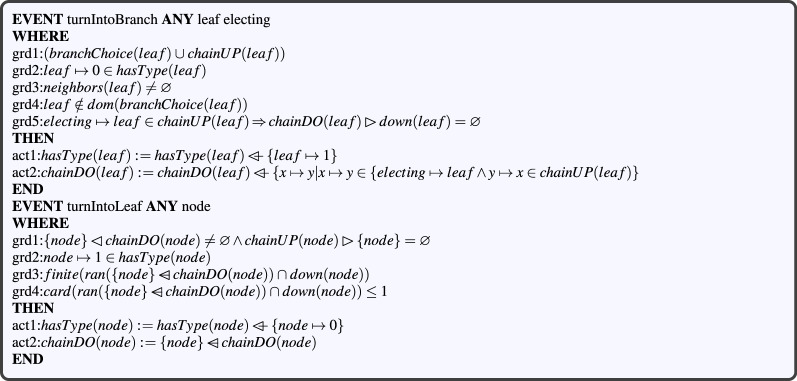
Node type update events.

Provided that the following conditions (expressed as a guard) are satisfied:

Event *turnIntoBranch* allows turning a leaf node into a branch node when it has been elected by at least one of its neighbors (see grd1, Fig. 11). This type changing occurs after receiving a hello packet from a neighbor. Guard *grd*3 checks CBL property *req*6 which states that self-election is not authorized (a node cannot be elected by itself). Event *turnIntoLeaf* allows turning a branch node into a leaf when the latter overtakes its electing downstream branch node after updating the node’s position (see Fig. 11). Guard *grd*1 defines a precondition of this type changing according to rule *TCR*3.

## Discussion and methods

The methods adopted during the validation of the *CCM*4*CBL* model rely on a two-step verification approach: validation of the model by animating it using the ProB model-checker, and proving its correctness by discharging proof obligations.

**Figure 12:**
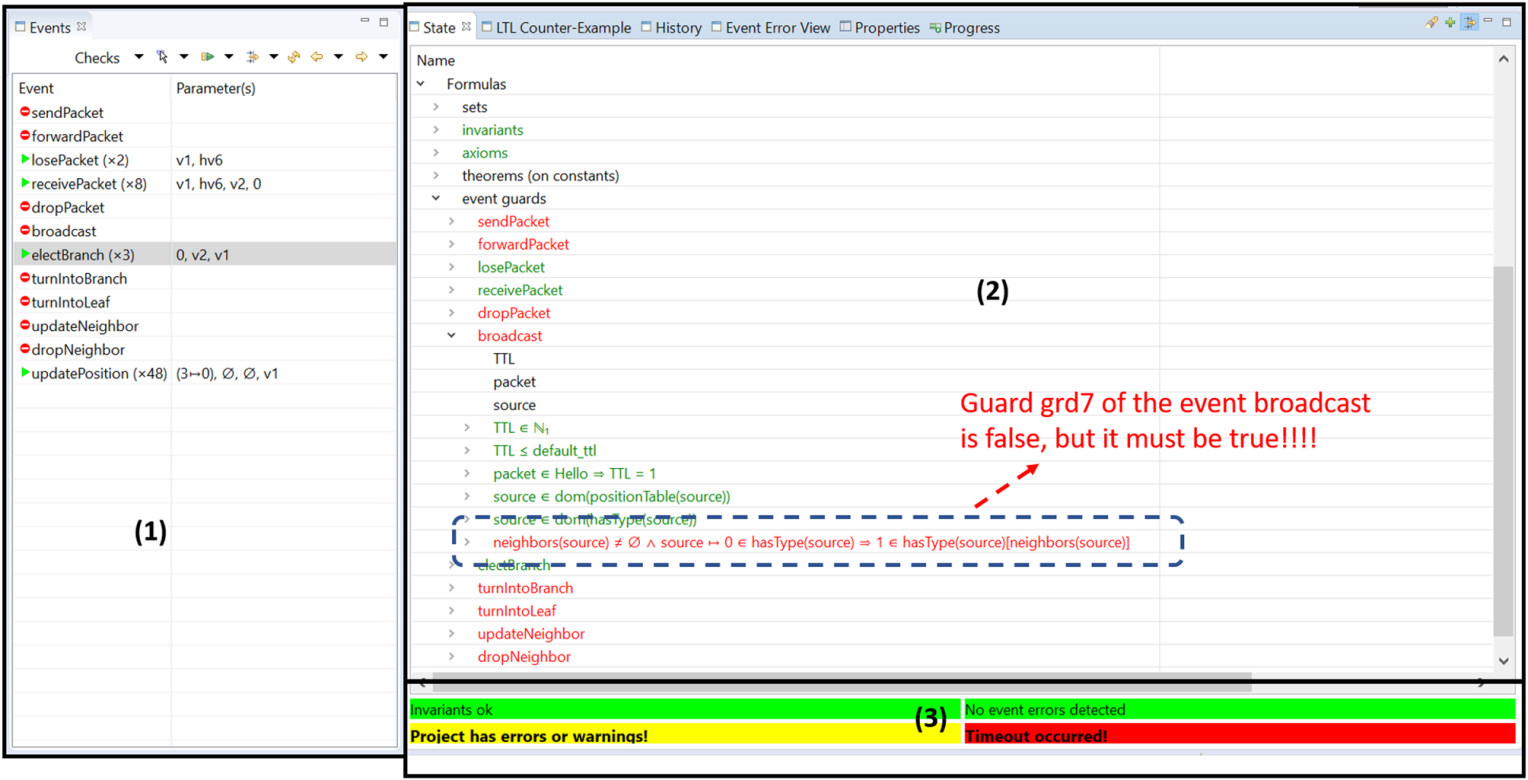
ProB animation window of the *CCM*4*CBL* model.

### Animation-based validation

The ProB^28^ animation tool allows both the validation of the requirements, and the detection of errors in order to fix them, before starting the proof phase which can be long and complex. It cannot be performed on an abstract Event-B specification, and requires a concrete model. For that reason, we created a new $CBL\_m3$ machine by extending the $CBL\_m2$ machine. This machine does not introduce new events or variables. It sees a concrete $CBL\_c3$ context, which is an extension of the $CBL\_c2$ context. New constants and axioms are defined in this extended context for the concretization of all the sets and functions introduced in the contexts of the proposed *CCM*4*CBL* model. Figure 12 depicts the ProB animation window which is composed of three main parts. The first part (1) describes event triggering and constraint checking. The second part (2) presents the status of the model. The last part (3) allows signaling potential invariant violations and specification errors. After setting up the animation context, only the initialization event is enabled. After that, *updatePosition* and *broadcast* events are successfully activated. Once a hello packet is broadcast by a node, the *receivePacket* and *losePacket* events are enabled. Event *updateNeighbor* is triggered several times after executing *receivePacket*. Regarding the animation scenario Fig. 12 shows, we note that *broadcast* and *turnIntoBranch* events are disabled after executing both events *updateNeighbor* and *electBranch*. No node will be turned into a branch and the clustering scheme cannot proceed. This situation suggests that a given node is not able to broadcast hello packets to inform the neighbors of its branch choice. It is caused by guard *grd*7 (see Fig. 12), which states that one of the neighbors of each leaf shall be a branch. In order to solve this issue, we modified the related guard:

$$\begin{aligned} neighbors(source)\ne {}\mathord \varnothing {} \wedge {}source\mapsto {}0\in {}hasType(source) \mathbin \Rightarrow {}\{source\}\mathbin \lhd {}branchChoice(source)\ne {}\mathord \varnothing {}. \end{aligned}$$

We used the ProB counter-examples as guides to rectify our model and trace back the specification errors, which could have caused the prover failures. The performed rectifications concern invariant violations and both guard and action alterations. We validated our model based on representative scenarios, including several cases which had not been treated previously.

### Correctness of the model by discharging proof obligations

In order to verify the model during its design process, proof obligations (POs) are discharged in a way guaranteeing that:

- Model initialization leads to a state where the invariant is valid.
- When the machine is in a state where the invariant is valid, every enabled event leads to a state preserving this validity.
- The concrete events can only occur in the circumstances in which the abstract events occur.
- The occurrence of any concrete event implies an occurrence of the related abstract event in such a way that the state verifies all related invariants.

**Figure 13:**
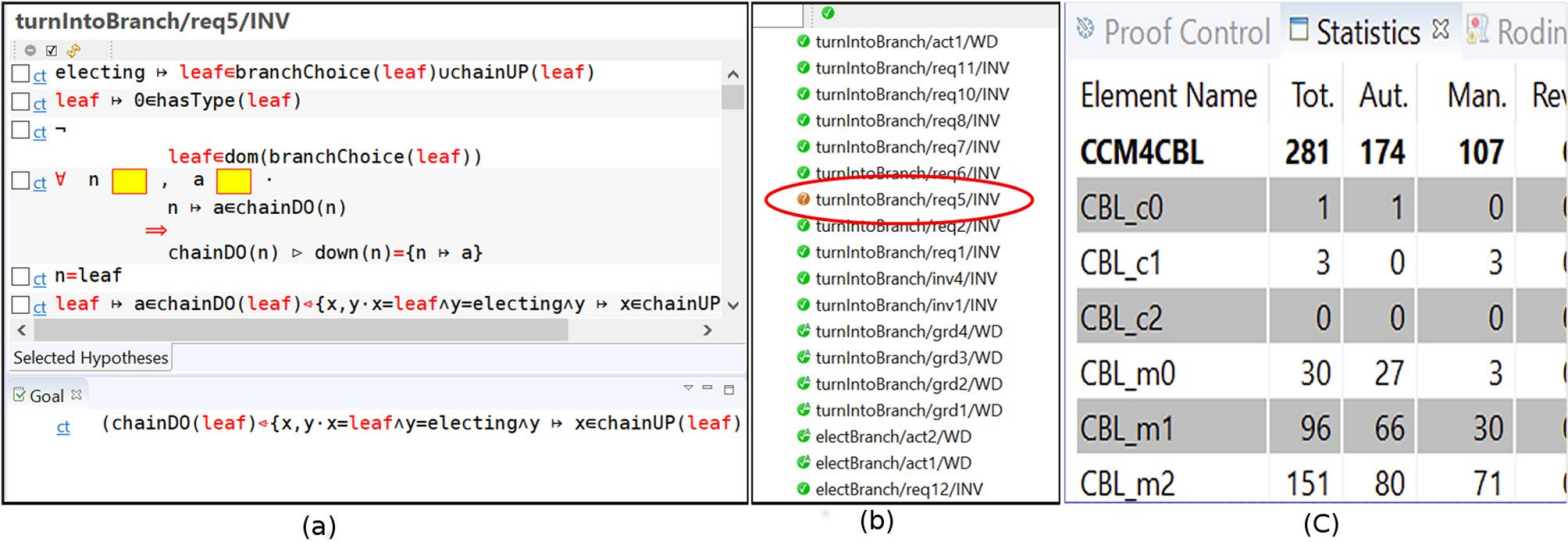
Examples of proof obligations and proof statistics in Rodin.

POs refer to the proofs applicable to an Event-B model. Figure 13 illustrates examples of proof obligations. Those discharged are marked with , while those undischarged can be recognized thanks to the symbol . The POs which are dischared automatically also show an “A” letter. Some proof obligations need interactive discharging by users, when the automatic prover cannot perform it (Fig. 13c shows the repartition between automatically and manually dischared POs in our model). For example, using the cardinal operator implies a finite set as operand, thus making more difficult the discharging of proof obligations. As well, the universal ($\forall {}$) and existential ($\exists {}$) quantifiers may be sources of concerns for the instantiation of the quantified hypothesis. In the proposed model, an example of such a PO is *turnIntoBranch*/*req*5/*INV*, which ensures that the *turnIntoBranch* event preserves the *req*5 invariant of the $CBL\_m2$ machine. As depicted in Fig. 13a, the sequent of such a PO is unproved due to a lack of hypothesis. For that reason, we modified the *turnIntoBranch* event, shown in Fig. 11, by adding the following new guard, which let to success:

$$\begin{aligned} electing\mapsto {}leaf\in {}chainUP(leaf) \mathbin \Rightarrow {}chainDO(leaf)\mathbin \rhd {}down(leaf)=\mathord \varnothing {}. \end{aligned}$$

## Conclusion and prospective work

In this paper, we have proposed an approach using Event-B to validate the properties and rules of a VANET routing protocol. Targeting the CBL clustering scheme, a correct-by-construction model *CCM*4*CBL* is proposed for that purpose as a proof of concept. This model includes three abstraction levels. The first one is an initial specification containing the basic functions of any network routing protocol. The second level introduces specific concepts of the VANET environment such as spatial relations between the vehicles according to their positions, vehicle movement, and management of both routing tables and vehicle communications. At this step, the proposed model can be reused for modeling any VANET routing protocol. The last level formally defines the specific properties and rules of the CBL clustering scheme regarding VANET organization. The proposed model is gradually verified using proof obligation mechanisms offered by the event-B method and finally validated using the ProB animator to repair several behavioral errors. These processes are illustrated thanks to concrete examples. In our future work, we will target the coupling of this formal approach to others, such as discrete event modeling and simulation, in order to enhance VANET protocols verification and evaluation, particularly regarding scalability and security properties.

## Data Availability

The code source is available.
